# Modeling the Impact of Alternative Immunization Strategies: Using Matrices as Memory Lanes

**DOI:** 10.1371/journal.pone.0141147

**Published:** 2015-10-28

**Authors:** Wladimir J. Alonso, Maia A. Rabaa, Ricardo Giglio, Mark A. Miller, Cynthia Schuck-Paim

**Affiliations:** 1 Fogarty International Center, National Institutes of Health, Bethesda, Maryland, United States of America; 2 Origem Scientifica, Epidemiology and Modeling, Sao Paulo, Sao Paulo, Brazil; 3 Clinical Research Unit, Oxford University, Ho Chi Minh City, Vietnam; 4 Department of Economics, Federal University of Santa Catarina, Florianopolis, Santa Catarina, Brazil; University of Waterloo, CANADA

## Abstract

Existing modeling approaches are divided between a focus on the constitutive (micro) elements of systems or on higher (macro) organization levels. Micro-level models enable consideration of individual histories and interactions, but can be unstable and subject to cumulative errors. Macro-level models focus on average population properties, but may hide relevant heterogeneity at the micro-scale. We present a framework that integrates both approaches through the use of temporally structured matrices that can take large numbers of variables into account. Matrices are composed of several bidimensional (time×age) grids, each representing a state (e.g. physiological, immunological, socio-demographic). Time and age are primary indices linking grids. These matrices preserve the entire history of all population strata and enable the use of historical events, parameters and states dynamically in the modeling process. This framework is applicable across fields, but particularly suitable to simulate the impact of alternative immunization policies. We demonstrate the framework by examining alternative strategies to accelerate measles elimination in 15 developing countries. The model recaptured long-endorsed policies in measles control, showing that where a single routine measles-containing vaccine is employed with low coverage, any improvement in coverage is more effective than a second dose. It also identified an opportunity to save thousands of lives in India at attractively low costs through the implementation of supplementary immunization campaigns. The flexibility of the approach presented enables estimating the effectiveness of different immunization policies in highly complex contexts involving multiple and historical influences from different hierarchical levels.

## Introduction

Because not all scientific questions are amenable to experimentation and data is often lacking, attempts to understand the world often rely on simplified models of reality. Modeling has been indeed an essential part of enquiry in the natural, economic and social sciences. Where the objective is to understand and simulate the behavior of populations, several methods are available to present day modelers. On the one hand, micro-level models enable consideration of individual histories and the ways that individual entities (e.g. persons, disease vectors, cells) interact. This is the case, for example, of agent-based models, which take a bottom-up approach by simulating interactions of multiple independent entities to predict phenomena at larger scales. Examples include studies on population viability [1], animal movement and dispersal [2] and the containment of pandemics [3]. Agent-based modeling is an ideal tool for studying emergent properties, as their design allow for complex macro-behavior to emerge through the interactions of independent agents following a set of pre-defined rules [4]. Within this modeling perspective, the dynamics of relationships between the entities of a system are as important as the isolated characteristics of those entities.

Macro-level models, conversely, focus on average properties of systems and consider aggregates of entities with similar characteristics. Examples include population-based models of disease transmission [5], cellular differentiation [6] and economic models of optimal resource allocation [7]. Consider, for example, the concept of state-transitions, namely the probability of moving from one relevant state (e.g. physiological, economic immunological) to another. A remarkable application [8] of such modeling framework explained—with a simple nonlinear dynamic model—the pattern switching of measles infection after the introduction of large-scale vaccination: prior to vaccination campaigns, measles epidemics were cyclical (biennial), becoming highly irregular and of smaller magnitude following large scale immunization efforts.

However, model parsimony and simplicity do not come without costs. Commonly used macro-level state-transition models subdivide a population into discrete units (or compartments) corresponding to different states and often assume that the probability of moving from one condition to another does not depend on history (e.g. prior states or time spent in the current state). Because this assumption can be unrealistic, these models usually rely on the creation of states that take average histories into account by incorporating them in their definition [9]. For example, in epidemiological models of infectious disease, transmission history is taken into account by dividing a population into immune, susceptible, infected, and recovered or even more refined levels [10]. Although able to explain some of the observed emergent properties, this solution might be limited by the need to balance a potentially exceedingly large number of states with the risk of overlooking the contribution of relevant prior events. For instance, the immunological profile of a population (macro-level) and probability of infection can be affected by individual histories (micro-level) in several ways, including age and timing of immunization, the number of vaccine doses taken, individual risk factors, waning immunity, and individual social contact patterns.

In summary, there is a clear trade-off between modeling flexibility and parsimony: on the one hand, modelers might be tempted to rely on the simplest models that can reproduce some of the emergent properties observed in the real world, but with a very coarse resolution. On the other, models may become too complex if attempting to address many of the emergent properties of systems simultaneously, hampering efforts towards model validation.

We present a framework that integrates the relative simplicity of macro-level compartmental models with the possibility of (1) taking the granular history of all elements of a population into account at all times and (2) allowing for interactive agent-based behavior. The model is conceptually described in the following section, and its full specifications are available in the Model Formulation section of S1 File.

### Multidimensional matrices as memory lanes

Although the framework is potentially useful in other fields, here we use it to analyze the cost-effectiveness of alternative immunization strategies. We show the proposed framework is (1) flexible enough to incorporate a diverse set of complex immunization strategies available to present day health authorities, and (2) realistic enough to reproduce aggregated (emergent) impacts of vaccination strategies within a population. To this end, it merges the explanatory power of dynamic compartmental models with the richness of detail brought by cohort-based modeling.

**Fig 1:**
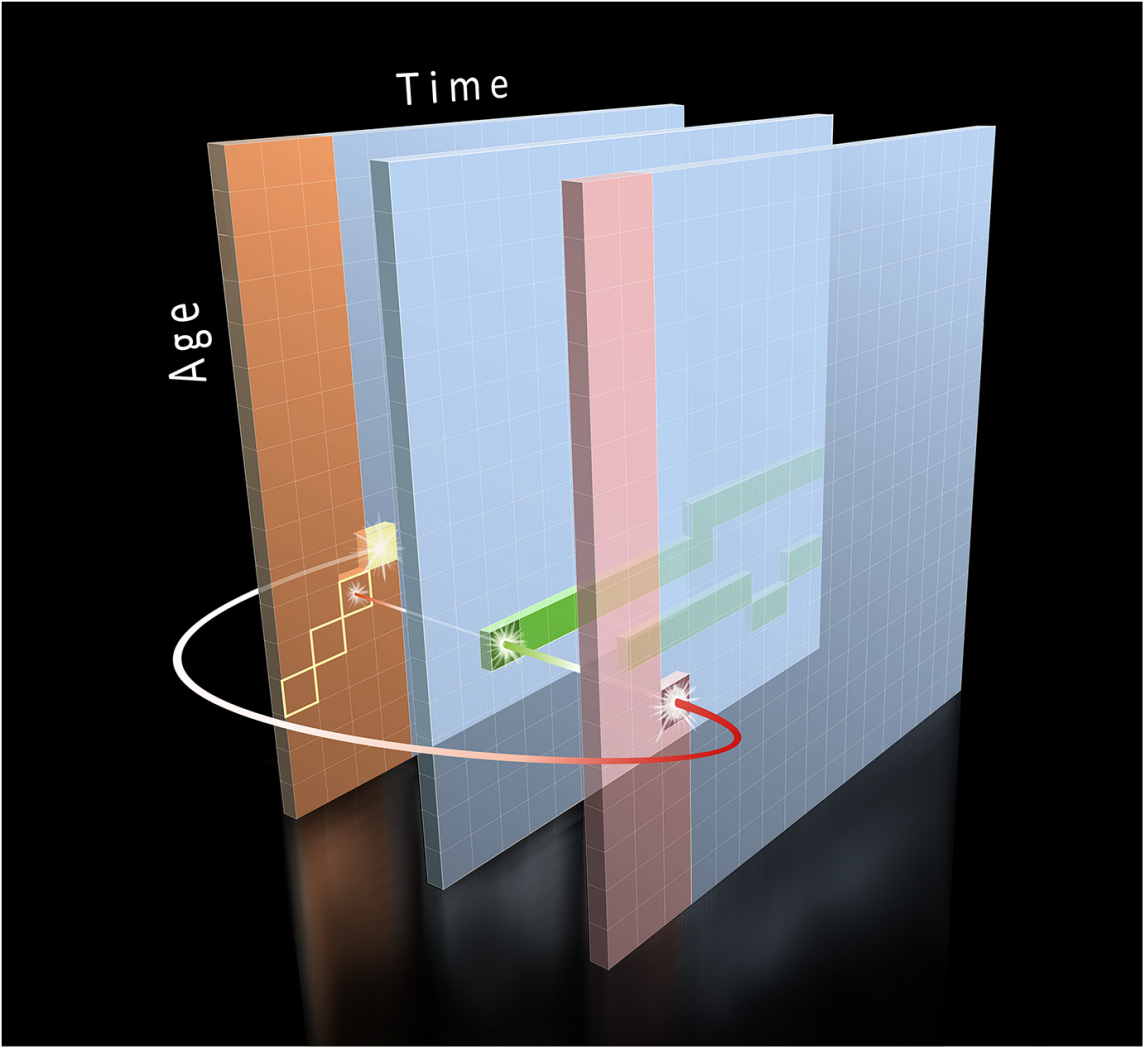
Representation of the dynamics whereby matrices are populated with data in the memory lanes model. The arrow departs from a cell with information on the proportion of susceptible individuals from a cohort at any time (e.g. year), and updates the information on immune individuals in the population after collecting information on any events (e.g. vaccination—mid grid, and infection—rightmost grid) happening at that time for that age cohort. Every time that a cycle is completed time advances by 1 unit (here 1 year) and each cohort ages accordingly by 1 unit of time—therefore, age cohorts move diagonally in the matrix. This process is repeated for all age cohorts and years until the matrix is completely populated. In real immunization modeling exercises (such as the one applied here for modeling the dynamics of measles), the number of matrices should be much larger to accommodate information such as waning immunity, herd immunity, migration, social patterns of contact, spatial distances, etc.

The framework can be thought of as a multidimensional matrix that serves as a memory warehouse of events, parameters, and states associated with a population (a group of discrete entities) at all times. This matrix is composed by a series of bidimensional grids indexed by time and age. Each bidimensional time×age grid represents information about one state or type of event (that is, a compartment). The conceptual framework is illustrated in Fig 1 using the basic structure of an epidemiological model of disease transmission, where changes in the immunological profile of a population are modeled over time. A graphical description is preferred to visually depict the intuitive nature of the approach, which mimics age-structured systems of difference equations. Technical notation is left to the Supporting Information (Model Formulation in S1 File). Grids are represented by time in the horizontal axis and age in the vertical axis. Intersections of age and time positions are referred to as cells, which are populated with data (model inputs) and the outputs of the modeling process. States represent the third dimension of this matrix. Here, each cell stores information on the proportion of an age stratum at a specific state (e.g. vaccinated, infected, susceptible) at each time point. In computational language, the matrix is equivalent to a three-dimensional array ‘A’ of the form A_[time][age][state]_. For example, if 40% of a population of 1000 children aged 1–4 years were immune to a disease in 2010, A_[2010][1-4yo][immune]_ = 40% (or 400).

Having bidimensional grids structured by time and age enables the preservation of the history of states and events of interest of an entire population as it ages. This stored memory can be then used recursively as input in subsequent cycles. Because cells are defined by two discrete time dimensions in every modeling cycle, age cohorts move diagonally, where the horizontal step represents the passage of time and the vertical step represents the corresponding aging. Depending on the rules determining state-transitions at the end of each cycle, a proportion of entities can either move diagonally from position (time, age) to position (time+1, age+1) within the same state or into another state if a state-transition occurs.

Rules defining state-transition probabilities are based not only on past history (e.g. time since last vaccination) but also on interactive, dynamic population parameters. For instance, infectious disease transmission can be halted depending on the proportion of immunes in a population, a phenomenon known as herd immunity ^9^. Under this framework, information on the proportion of immunes (determined from factors including vaccine efficacy, number of vaccine doses, and previous infection history) is available at all times for all age strata at the appropriate matrix positions, enabling the estimation of the extent to which herd immunity will affect age-specific infection probabilities. Similarly, the probability of infection incorporates socio-behavioral information such as age-specific patterns of contact (which can be stored in a separate age×age bidimensional grid). Disease infectivity can thus be calculated dynamically depending on historical social networking patterns.

We also considered historic information for specific periods or age groups in a customized manner. For instance, use of an input grid with vaccine numbers administered for each age group at each year enables considering years when immunization campaigns targeted a broader age range, when vaccines were not administered or age-specific coverage levels (mid grid, Fig 1). Additionally, events and state-transition probabilities can be either deterministic (the same initial conditions always produce the same outcomes) or stochastic (random factors are included and outcomes have different probabilities). For example, difference equations can be used to calculate the proportion of infected individuals based on population structure (e.g. age-specific contact rates) and disease properties (e.g. incubation period, infectivity). Models may also include stochastic simulations such as the likelihood that an infected person recovers or that a susceptible person meets an infected individual. A single model may even include both deterministic and stochastic elements, each employed for specific events. It is worth noting that it is possible to represent the modeling output as a complex system of Partial Differential Equations (PDE). Conversely, Agent Based Models (ABM) can also represent any system of PDE. The framework we describe here connects the best of these two worlds (PDE and ABM) by allowing researchers to preserve the entire history of all population strata and enable the use of historical events, parameters and states dynamically in the modeling process.

This framework is applicable in areas as diverse as the medical, social, economic, and biological sciences, but to demonstrate its workings we apply it to examine the cost-effectiveness of alternative immunization policies against measles in developing countries. Measles remains the leading cause of vaccine preventable childhood mortality in many countries [11] and the risk of resurgence of measles in Africa is particularly high, reinforcing the importance of timely and effective immunization policies. Additionally, measles dynamics has been exhaustively modeled [12–16], allowing validation of modeling outcomes.

### Modeling immunization strategies to fight measles in the developing world

Measles is highly transmissible with approximately 90% of susceptible people coming into close contact with an infected person becoming infected. Even in highly vaccinated populations, episodic outbreaks can still occur due to the immigration of infected individuals, social clustering of susceptible people, waning immunity, and high birth rates [17–18]. Very high population immunity levels must therefore be constantly maintained at national and sub-national levels to sustain measles elimination in the long term. To help achieve this goal, the World Health Organization (WHO) recommends that countries provide all children with a second opportunity for measles immunization either through a second routine dose of measles-containing vaccine or periodic large-scale supplementary immunization activities (SIAs) targeting a wide age range.

**Fig 2:**
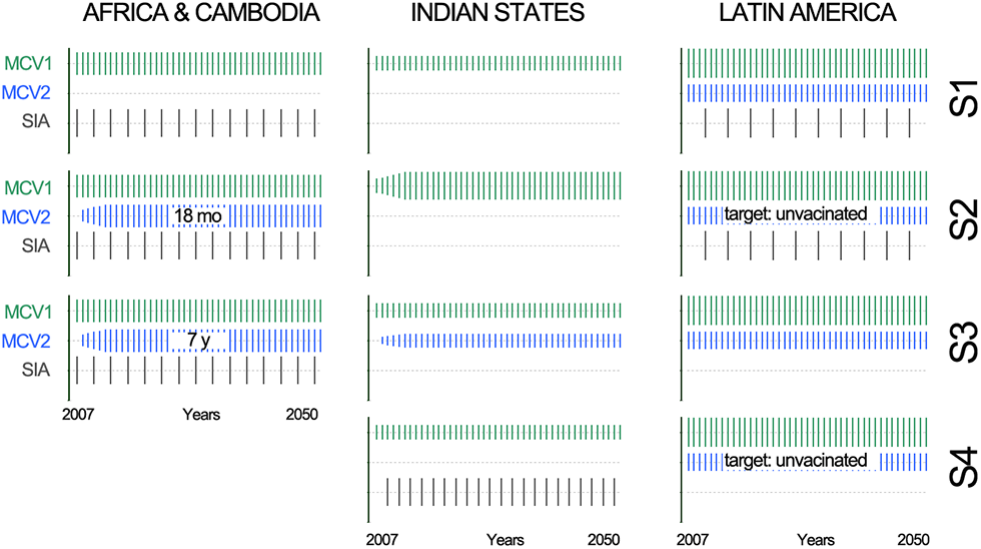
Measles immunization scenarios (Status quo, S2, S3, S4) simulated up to 2050. Vertical bars are proportional to coverage levels. First (MCV1) and second (MCV2) routine doses and supplementary immunization campaigns (SIAs) are represented in green, blue, and grey, respectively. Scenarios were selected by the WHO Strategic Advisory Group of Experts (SAGE). Dependence between MCV1 and MCV2 indicates the % of unvaccinated individuals targeted to receive MCV2. Indian States: Status quo: MCV1 dose at 9 months at current coverage (46%, 90%, 74%, 86%, and 95% in Bihar, Karnataka, Maharashtra, Orissa, and Tamil Nadu, respectively); S2: MCV1 coverage improved to 90% over 5 years; S3: status quo plus inclusion of MCV2 at 18 months, improving from 50% to 97% of MCV1 over 5 years (dependence between doses = 25%); S4: inclusion of SIAs from 2009 onwards targeted at ages 9 months to 5 years with 90% coverage. Africa and Cambodia: Status quo: MCV1 (coverages of 73%, 73%, 51%, 85%, 95%, and 79% in Cameroon, Democratic Republic of Congo, Equatorial Guinea, Ghana, Rwanda, and Cambodia, respectively) and SIAs every 2–4 years (target age: 9 months to 5 years, 90% coverage); S2: status quo and MCV2 at 18 months with coverage improving from 50% to 100% of MCV1 over 5 years (dependence between doses = 25%); S3: status quo and routine MCV2 to 7 year olds, MCV2 coverage improving from 50% to 100% of MCV1 over 5 years (dependence between doses = 25%). Latin America: Status quo: MCV1 (at 15 months) MCV2 (to 7, 4, 4, and 6 year olds in Costa Rica, El Salvador, Paraguay, and Mexico, respectively) at existing coverage (MCV1 and MCV2 coverage in Costa Rica, El Salvador, Paraguay, and Mexico, respectively: 89% and 94% of MCV1; 98% and 94% of MCV1; 88% and 66% of MCV1; 96% and 58% of MCV1) and SIAs implemented every 4 years (target age: 9 months to 5 years, 85% coverage in Costa Rica and 92% in remaining countries); S2: status quo and 100% dependence between MCV1 and MCV2; S3: status quo and elimination of SIAs; S4: status quo elimination of SIAs and 100% dependence between MCV1 and MCV2.

We modeled alternative immunization strategies aimed at accelerating measles elimination in 15 countries in Asia, Africa, and Latin America. Specifically, we investigated the consequences of increasing coverage of a single routine vaccine dose compared to the added value of introducing a second routine dose in the presence or absence of SIAs. We also examined the effectiveness of SIAs in countries that already employ two routine doses at various levels of coverage. The choice of countries and design of immunization scenarios (Fig 2) were conducted by the WHO Strategic Advisory Group of Experts (SAGE).

The ability to preserve and track the history of a population through the use of temporally structured matrices can be particularly useful where it is unfeasible to create exceedingly large state spaces or reduce the complexity of a system to a limited set of analytical equations. Each time×age grid represented one state (such as susceptible, vaccinated, infected, immune, and dead). Because the temporal resolution of data available for most low income countries is annual, time was represented in annual steps from 1980 to 2050. Age was structured by month for infants <1 year and by year for older ages. Country-specific immunization information from 1980 to 2008 comprised historic records of vaccine coverage by age and year and, from 2009–2050, represented the alternative hypothetical immunization scenarios shown in Fig 2. Country-specific demographics (population estimates, birth rates, changing mortality, and life-expectancy by age and year) were also available for 1980 to 2008 and represented country-specific projections for the remaining years up to 2050. Previously established epidemiological and cost parameters (socioeconomic status, historical vaccine coverage, age-specific vaccine efficacy, case-fatality ratios, and vaccine costs; Model Formulation in S1 File) were used to determine the probability of state-transitions and the cost-effectiveness of each immunization strategy.

**Fig 3:**
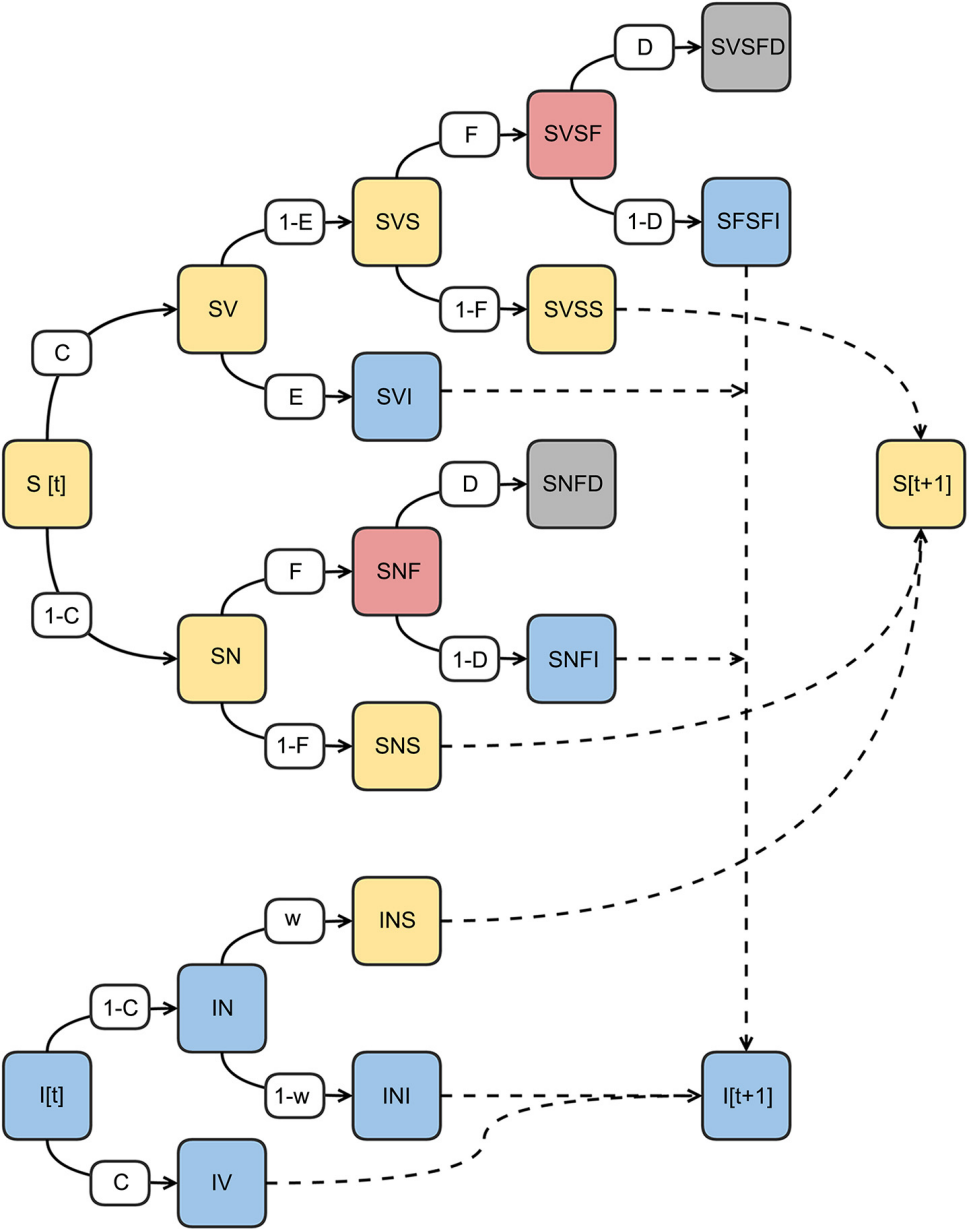
Diagram summarizing the events each age cohort can experience in an annual cycle (it can be also understood as the outcomes an individual may experience each year). The cycle starts (left) and ends (right) with the proportions of susceptible and immune individuals. Events (vaccinations, infections, mortality) during the cycle change the proportions exported for the next cycle and generate intermediate outcomes of interest (proportions of cases and deaths). Each box represents a matrix of 104 age-groups × 71 years. Probabilities are depicted in the small white boxes: C: Vaccine coverage, E: Vaccine efficiency, F: Force of infection, D: Case fatality ratios, and W: waning Immunity probability. The large colored boxes are compartments, and sequences of letters represent sequences of events (e.g. SVSFI blue box: proportion of susceptible individuals S who were vaccinated–SV–but remained susceptible–SVS–were next infected–SVSF–and became immune SVSFI). N stands for non-vaccinated.

Each cycle started by determining the proportion of susceptible and immune individuals at each age (Fig 3) based on their corresponding proportions at the end of the previous cycle and the effect of maternal immunity on new (incoming) birth cohorts. Given the high population turnover in the countries studied and long period modeled, model outcomes were not affected by assumptions on existing proportions of susceptible and immune individuals in the first cycle (Model Formulation in S1 File). Next, vaccine coverage determined age-specific proportions of individuals vaccinated. Vaccine efficacy was then used to calculate the proportion of the vaccinated population that became immune. Individuals still susceptible could be infected depending on their age, country-specific measles parameters, social contact rates, and the proportion of susceptible individuals in the population. In the latter case, the likelihood of infection was determined dynamically, as the immunological profile of the population (proportion of susceptible individuals in each age group) in the previous cycle was used recursively as an input that established a dynamic force of infection.

The model also considered social contact patterns and the possible immigration of infected individuals (a stochastic input parameter that was country-specific), which added to the force of infection. Finally, age-specific case-fatality rates determined the proportion of infected individuals who died or became immune. Age-specific proportions of immune and susceptible individuals at the end of the cycle were then used as initial proportions in the subsequent cycle (Fig 3). In computational terms, the framework worked by initially looping through all age groups in the first cycle, filling in blank cells with the proportion of each age group at each state in that cycle. When computations were complete for the entire period, proportions were translated into absolute numbers by using age-, year-, and country-specific demographics. Because demographic information already included realistic data and projections of changes in birth rates and in unrelated causes of mortality, no further assumptions about these parameters were required. All model assumptions, algorithms, epidemiological, and cost parameter data sources and manipulations are described in detail in the Supplementary Data.

## Results and Discussion

**Fig 4:**
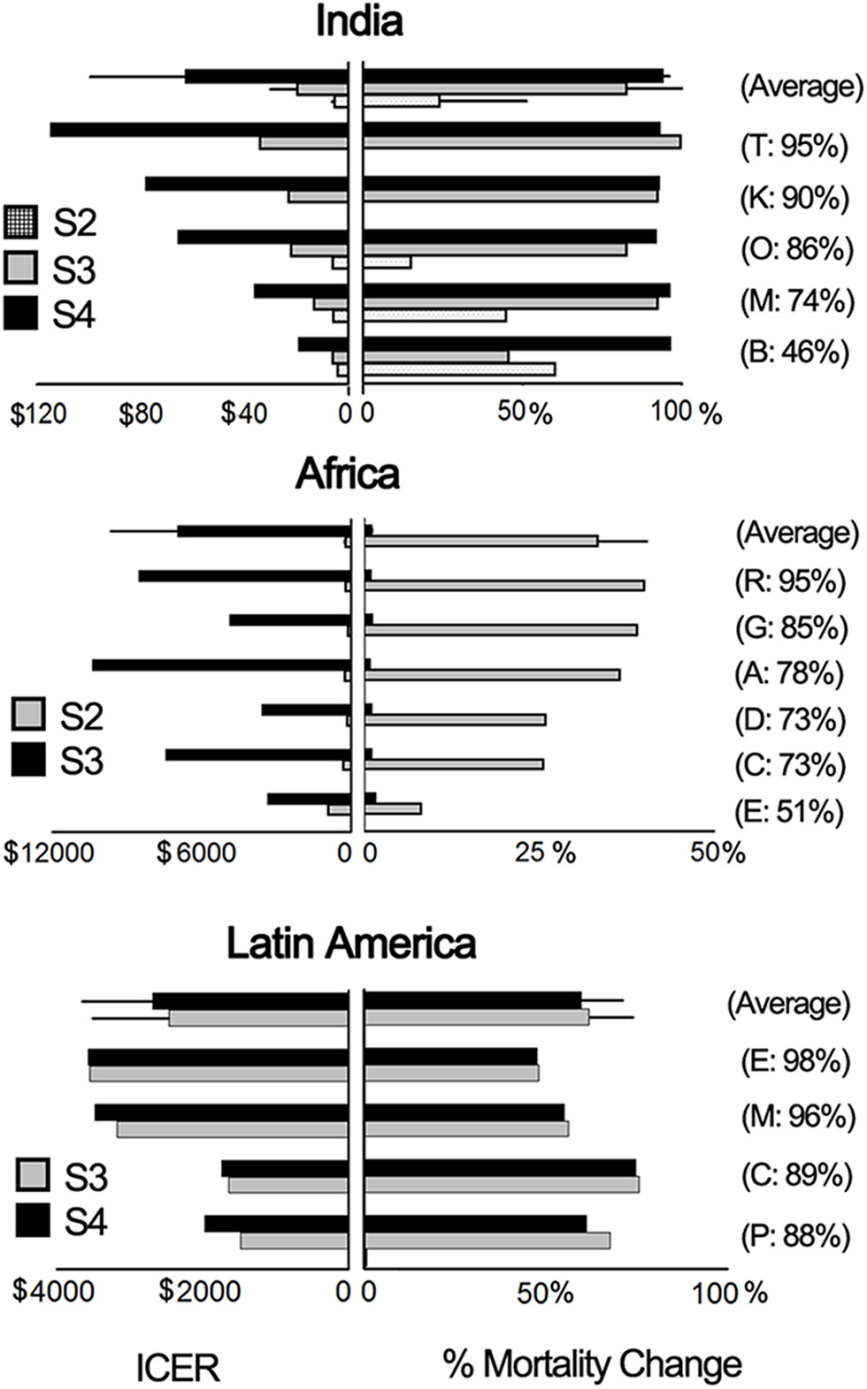
Cost-effectiveness (panels on the left represent incremental cost-effectiveness ratios (ICERs) in US$ per DALY averted) and percent change in mortality (panels on right) of alternative immunization strategy (S2, S3, S4) relative to baseline status quo immunization scenarios (S2/S3/S4 are described in Fig 2). For Latin America, ICERs represent the cost saved per DALY averted and mortality change as percent increases in mortality. For the other regions, mortality change represents the percent reduction in mortality. Bars are ordered according to MCV1 coverage.

The cost-effectiveness of the alternative strategies simulated (Fig 2) depended on the properties of baseline immunization scenarios (Fig 4), chiefly pre-existing vaccine coverage rates. In India (where a single vaccination opportunity is administered at an early age), effective improvements in population immunity were achieved by all strategies (increasing first vaccine coverage, adding a second routine dose, or implementing SIAs). However, implementing SIAs resulted in the greatest reduction in disease burden at attractively low costs. Because SIAs target a wide age range, this is an effective strategy where a large proportion of the population is still susceptible. In the absence of SIAs, raising first dose coverage was the most effective immunization strategy in regions with low coverage, whereas adding a second routine dose was more effective only where coverage is already high. In the African countries and Cambodia, where birth rates are high, it was more effective to provide a second routine dose at 18 months rather than to school-age children. Finally, the elimination of SIAs in Latin America should be considered only where very high coverage levels can be maintained at national and district levels.

Modeling outputs consistently recaptured long-endorsed policies in measles control, showing that where a single routine measles-containing vaccine is employed with low coverage, any improvement in vaccine coverage is more effective than adding a second dose (Fig 4). The model also identified an opportunity to save thousands of lives in India at an attractively low cost through SIAs–an important finding considering that approximately half of all global measles deaths are concentrated in this country [19]. These results were robust to sensitivity analyses including a wide range of epidemiological and cost parameters (see S1 File—Parameters, data sources and manipulation). These conclusions were also insensitive to changes in the assumptions on the transmission dynamics of measles (Supplementary Data in S1 File) and should be helpful in informing ongoing policy debates over the use of SIAs [20–23].

Model-based analyses can aid in the development of optimal immunization policies, enabling simulation and assessment of alternative strategies and identification of key elements of successful programs. Given the inherent paucity of input data for most low-income countries, our modeling example was implemented at the expense of epidemiological detail. Although the consistency in the outcomes is reassuring, further refinement is possible. Because the temporal resolution of most of the data available is annual, and the purpose of the modeling effort to compare the overall effectiveness of alternative immunization strategies, the model did not account for sub-annual aspects of measles dynamics such as seasonality in transmission [10,24]. Additionally, we assumed that case fatality rates and vaccine costs per child remained constant as vaccination coverage scaled up. To the extent that these assumptions are violated, absolute estimates of cost-effectiveness are similarly affected, impairing the ability to compare the cost-effectiveness of controlling measles as opposed to other diseases.

The modeling application presented here can also expanded to include other relevant features such as spatial components. Instead of a single cubic matrix, it may incorporate multiple cubic matrices (each corresponding to one location). Transitions or movement from one location to another can then be modeled as a function of distance or attrition (e.g., flux of people, connectedness) between locations. For example, one could consider the location of infected individuals and their typical movement patterns, modeling the probability of infection based on distance from other locations and corresponding infection prevalence [25].

The framework we propose combines conveniences from different modeling perspectives. First, it integrates population- and individual-based models in several ways. Grid cells can represent either population strata or individuals, higher level phenomena that emerge from individual behaviors and histories can be incorporated into the modeling process in a dynamic way and, conversely, population dynamics can be fine-tuned by individual histories and events. The ability to preserve and track the history of a population through the use of temporally structured matrices can be particularly useful where it is unfeasible to create exceedingly large state spaces or reduce the complexity of a system to a limited set of analytical equations. Additionally, the rules governing the outcomes of interactions, state-transitions, and events can be determined both in a deterministic and stochastic way, as custom algorithms may be used independently at different modeling steps, giving the framework a high level of flexibility and wide scope of application.

Models are an inseparable part of scientific enterprise. We have proposed a discrete-time discrete state-space modeling structure that enables the integration of high levels of heterogeneity and resolution with macro-population modeling in an intuitive manner, through the use of temporally structured matrices that can consider a (theoretically) unlimited number of variables. These matrices serve as memory lanes that preserve the history of a population and enable its recursive use in the modeling process. The relative simplicity of the framework should make it attractive to aid researchers and decision-makers in estimating the effectiveness of alternative immunization strategies, as well as optimal courses of action in all fields where modeling is an essential part of enquiry.

## Supporting Information

S1 File Supporting Information. Model formulation, parameters, data sources, manipulations and tables A-K.
(DOCX)
