# Supplementary material for: Modeling the Impact of Alternative Immunization Strategies: Using Matrices as Memory Lanes
Source: PLoS One. 2015 Oct 28;10(10):e0141147. doi: 10.1371/journal.pone.0141147 (PMC4624994; doi:10.1371/journal.pone.0141147)
Supplement: S1 File — Model formulation, parameters, data sources, manipulations and tables A-K. (DOCX) [file pone.0141147.s001.docx]

**Supporting Information (S1 File)**

## Model formulation

The modeling cycle is depicted in Figure 3 (main manuscript). Each cycle started with a proportion of susceptible and immune individuals at each age group, based on previously existing proportions, on the age-specific effect of maternal immunity (table A) on new birth cohorts, or on the immunity level assumed as initial condition for the dynamic system.


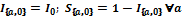

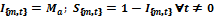


where stands for the share of immunes in a cohort defined by the age at the moment , is the immunity assumed at the initial simulation step, stands for the susceptible share of that cohort, indicates that this cohort is still under maternal anti-body immunity, and is the maternal immunity assumed for the age .


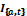

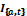

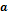

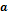

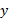

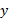

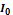

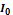

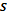

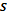

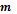

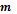

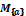

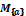

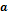

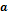


Susceptible individuals could be vaccinated or not depending on coverage .


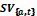

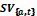

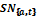

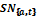

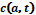

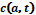

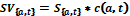

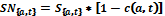


Vaccine efficacy  **is** determined as the proportion of vaccinated individuals who became immune.


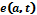

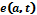

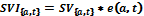

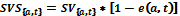


Individuals still susceptible could also be naturally infected, depending on a country- and age-specific force of infection that was modulated by the proportion of susceptible individuals in the population,


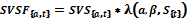

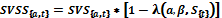

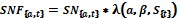

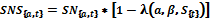


where stands for the share of the cohort infected after vaccination, and the share of unvaccinated infected. Similarly, and represent the shares of vaccinated and unvaccinated individuals who did not become infected, respectively. The force of infection depends on a inter-age group social contact patterns **,** and on the current overall state of susceptibility in the population . Additionally, we also tested for a country-specific (based on factors as income and remoteness) reduction in the force of infection in cases when her immunity threshold has been achieved. These three additional parameters for the force of infection are further addressed in the remaining of this Supplementary Material.


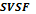

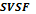

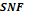

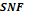

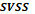

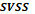

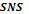

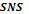

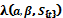

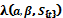

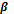

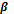

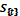

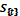


After infection, individuals can die depending on the country- and age-specific case fatality ratios ,


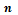

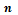

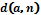

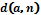

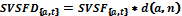

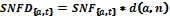


or recover from disease becoming then immune to it.


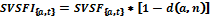

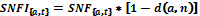


Vaccines had no effect on immune individuals, but the proportion of immunes who were vaccinated was stored to evaluate strategies targeting unvaccinated individuals.


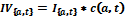

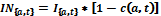


Intermediate outcomes of interest (cases and deaths ) can also be represented.


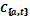

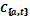

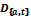

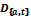

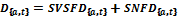

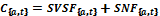


Finally, at the end of each cycle, the proportion of susceptible individuals at each age and time equaled the sum of uninfected unvaccinated individuals and vaccine failures. Measles casualties were next discounted from the population.


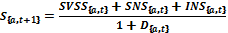

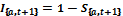


Bidimensional 104 age × 71 year (1980-2050) matrices stored all proportions for each of state. When all epidemiological computations were complete proportions were multiplied by the corresponding demographics (or projections). This procedure ensured that realistic fertility and mortality rates were appropriately took into account. For example, had there been substantial unrelated mortality for an age group in one year, the high number of deaths would led to a lower absolute number of susceptible, immune and infected individuals in that year.

As in Grenfell et al. [1] we used a catalytic model whereby the force of infection acting on an individual of age at time was an increasing function of the proportion of the susceptible overall population . Transmission to age class from age class also depended on their rate of contact, such that


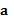

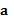

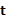

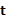

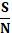

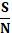

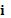

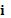

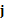

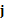

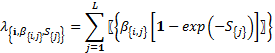


where is population specific longevity, is population size and the rate at which a susceptible individual from age class is infected by age class individuals. Here, we chose to model the force of infection as a function of the proportion of susceptible individuals because the model cycle is annual (had the model considered exclusively the disease cycle, a force of infection based on the number of infected people would be more appropriate). Because contact rate patterns are not available for low income countries, they were based on a large-scale study of epidemiologically relevant social contact patterns in eight European countries [2]. This large dataset is likely to represent an improved parameterization of contact rates compared to proxy measures of contact or WAIFW (who acquires infection from whom) matrices. Age-specific contact patterns were adjusted for each country, such that


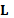

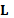

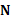

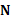

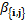

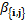

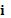

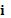

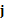

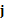

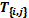

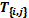

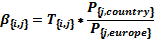


Where


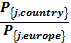

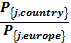


is the country’s population size in age group relative to the average population size of that age in the European countries.


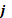

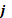


To further explore the robustness of our projections we simulated two additional transmission modes: (1) static transmission, where country income (high or low) was used as a proxy for household structure, determining age- and country-specific fixed forces of infection (Table A) and (2) semi-dynamic transmission, where age-specific force of infection (adjusted for income) was reduced when herd immunity was achieved in the population between 1 and 20 years of age (i.e. after maternal immunity waned and before adulthood, when measles transmission is negligible). The reduction in the force of infection followed a sigmoid function ranging from 89% to 96% (interval of possible herd immunity thresholds, as reported for measles; [3, 4]). To account for the possibility of importation of measles cases we also derived a stochastic factor from the average proportion of cases in the susceptible population of American countries between 1996 and 2006 (only countries with estimated susceptibility lower than 6.7% were included; Table A). The number of infected immigrants at any time was calculated separately for remote and non-remote countries (Table A).

## Parameters, data sources and manipulation

Immunity: an overall immunity of 93.3% before 1980 was assumed, as it approximates the herd-immunity threshold for measles [3-5]. The impact of cohorts from 1980 decreased with population turnover. Since the analysis looked at outcomes in the young population from 2008 onwards, results were virtually insensitive to initial conditions.

Vaccine coverage: estimates of country-specific coverage and schedule data for MCV1, MCV2 and SIAs were provided by WHO officials (Strebel, P.; Dabbagh, A., Gacic-Dobo, M.; personal communication in 2008). No MCV1 data were missing for any country, so missing data were treated as 0% coverage. MCV2 coverage data represented the best estimate reported to the WHO by each country. Although the MCV2 coverage dataset was incomplete, available data were used to best reflect the immunization activities of each country in the calculation of country-specific immunization profiles. Missing data were treated as 0% MCV2 coverage. Only confirmed SIAs containing information on target age and percent of the target population reached were included in the analysis. Campaigns implemented in the same country and same year were combined. For the Indian states, state-specific routine MCV1 and MCV2 coverage data from 1980 to 2005 were extracted from the WHO MSP tool [6].

Dependence between doses: we investigated economic benefits and extent of gains in population immunity when seeking to vaccinate unvaccinated individuals. Dependence for MCV1 and MCV2 ranged from 0% to 100%. When it was 100%, those who had missed the first dose were the first to receive a second routine dose. When it was 50%, 50% of non-recipients of the first dose were targeted to receive the second dose. With 0%, vaccines were administered randomly.

Waning immunity: we dealt with waning immunity by means of two additional input parameters, one for natural immunity (after infection) and another for vaccine obtained immunity. These input parameters represent the maximum in years that immunity wanes completely following a sigmoid function from the start point (immunization time) to the end point (maximum time in years immunity can possible last). We assume that (1-93.3%) of the vaccine obtained immunity wanes within 40 to 80 years. With respect to maternal antibodies, we assume in our model that it protects 100% of children between 0 to 5 months, 30% children from 6 to 8 months, 10% children between 9 to 11, and no maternal immunity is assumed further. We could observe periods of high overall susceptibility followed by aggressive outbreaks, as suggested [7] when the immunization brought by high coverage vaccination campaigns begin to wane in around 40 to 80 years.

Case Fatality Rates: country-specific case fatality rates (CFRs) for children aged 1 to 4 years were extracted from the WHO’s computer-based Measles Strategic Planning (MSP) tool [6]. Age-specific CFRs were estimated based on the assumption that children <12 months and 5-9 year old have, respectively, a higher and lower CFR than children aged 1 to 4 years. Children >10 years were assumed to have a CFR of 0.

### Demographic Data: data were extrapolated from the 2006 Revision of the World Population Prospects [8]. Age groups were considered as detailed in table A. This classification enabled simulating the effects of interventions at critical ages. WPP data were disaggregated using neonatal, infant, and under-five mortality rates to obtain morbidity and mortality estimates for each annual birth cohort. In addition, the first two years of life were further disaggregated to provide estimates of population size for the five sub-annual age cohorts. Because WPP data were provided in 5-year age groups, the data was disaggregated to determine the number of births and deaths within a year. To this end, we adjusted annual estimates by neonatal (NMR), infant (IMR) and under 5 mortality rates (U5MR), as well as the estimated population in each age group. For Indian states, comparable population data (5-year age groups) were only available from 1980-2025. NMR and IMR were calculated according to the proportional relationships between the U5MR, NMR, and IMR for India (NMR = 56.25% of U5MR; IMR= 75% of U5MR). State populations were then extrapolated to 2050 based on the trend observed over the previous 10 years. Annual birth cohorts for each Indian state were then calculated as for the remaining data.

## Income: country-specific gross national income (GNI) was obtained from the World Bank website. GNI was converted to U.S. dollars using the Atlas method, taking into account all production in the domestic economy (i.e., GDP) plus net flows of factor income (such as rents, profits, and labor income) from abroad. When GNI per capita was not available, country-specific GNI per capita was calculated as an average of estimated GNIs per capita for countries within the same WHO region and mortality stratum. Countries were categorized as high or low income based on a cutoff value of US$ 11,115 GNI per capita. Countries with GNI per capita estimates greater and lower than this threshold were considered to be high and low income countries, respectively. Income determined age-specific forces of infection within each country, as it was assumed to be a proxy for demographic and household structure [5].

###

### Cost Data: country-specific GNI data and vaccine costs listed by UNICEF and the U.S. Centers for Disease Control (CDC) were used to estimate the costs of routine vaccination and SIAs. In low income countries, the cost of MCV1 was estimated based on the UNICEF cost for each measles vaccine (US$ 0.19) and on the (log-transformed) relative income of the country compared to Burundi, the country with the lowest GNI in the database. Vaccine dose price was estimated as the vaccine cost multiplied by relative income. Total vaccination cost per person was the sum of the vaccine dose cost (representing 30% of total cost) and logistic and infrastructure costs (personnel, transportation, cold chain, capital, supplies, and miscellaneous costs), representing 70% of total vaccination cost. An additional 40% of the vaccine price ($0.19 × relative income factor × 0.4) was added to the total cost of vaccination per person to represent wastage [9]. MCV1 cost per person ranged from $0.71 to $2.15 in low income countries. In high income countries, the cost of MCV1 was estimated based on the CDC cost of the measles, mumps and rubella (MMR) vaccine, which in 2007 was US$16.01 (plus US$2.25 tax). The price of the measles component of the vaccine in the U.S. was assumed to be 1/3 of the cost of the MMR vaccine. Vaccine price per dose was estimated as the vaccine cost multiplied by the (log-transformed) relative income of each high income country relative to the U.S. Total vaccination cost per person was the sum of vaccine cost (31% of the total cost) and logistic and infrastructure costs (personnel, transportation, cold chain, capital, supplies, and miscellaneous costs), representing 69% of total vaccination cost without considering wastage rates [10]. An additional 12.5% of the vaccine price was then added to the total cost to represent wastage [9]. MCV1 cost per person ranged from $7.08 to $21.50. For both high income and low income countries, the cost of administering MCV2 was assumed to be include an additional 33% of personnel, transportation, cold chain, capital, supplies, and miscellaneous costs [10]. MCV2 cost per person, including wastage, ranged from $0.86 to $2.60 in low income countries and from $8.63 to $26.21 in high income countries. Supplementary immunization activities (SIAs) were calculated similarly to MCV1.In high and low income countries, vaccine price was assumed to be, respectively, 32% and 37% of the total cost of SIAs, with the remaining 68 and 63% representing personnel, transportation, cold chain, capital, supplies, and miscellaneous costs, without considering wastage rates [10, 11]. Wastage rates were assumed to be lower during SIAs and 10% of the vaccine price in both high and low income countries. The total costs of SIAs ranged from $0.53 to $1.62 per person in low income, and from $6.81 to $20.69 in high income countries. Vaccination costs were assumed to be the same for all Indian states, corresponding to total routine and SIA costs as based on the national GNI.

# Cost-effectiveness analysis: incremental cost-effectiveness ratio (ICER) of vaccination was measured as the ratio of the change in costs of vaccination (compared to baseline) against the benefits of vaccination, measured by the number of disability-adjusted life years (DALYs) averted [12]. The latter measure considers years of life lost (from country-specific life expectancy) from premature mortality due to the disease, and the loss of quality of life due to the average severity of disabilities caused by measles. We used a disability weight of 0.152 and duration of 0.4 years [13]. Costs were discounted at a rate of 3% [13]. Strategies considered cost-effective if ICERs were lower than the country’s per capita GDP.

# Sensitivity Analysis. In addition to analyzing sensitivity to changes in the dynamics of measles transmission, we examined the impact of variations in key epidemiological and cost parameters. For each region, the analyses were conducted for the country where differences among alternative strategies were lowest, namely where alternative strategies produced the most similar cost and effectiveness outcomes. To further increase the likelihood of detecting differences in the original conclusions of the modeling process, we used a multivariate analysis to examine the combination of parameters that produced best and worst case scenarios for each strategy. The following values were used: case-fatality rates (baseline value ± 50%), maternal immunity (baseline ± 50%), vaccine efficacy (baseline ± 10%), discount rate (0%, 3% and 7%) and vaccine dose cost (baseline ± 50%).

# Model implementation: All the computations were performed by the Decision Analytical Support for Strategies for Multiple Doses of Measles Vaccine tool [15], developed by some of the authors of this article and other collaborators from the Fogarty International Center to the World Health Organization (WHO). References

1. Grenfell BT, Anderson RM. *The estimation of age-related rates of infection from case notifications and serological data.* J Hyg (Lond), 1985. **95**(2): p. 419-36.

2. Mossong J, Hens N, Jit M, Beutels P, Auranen K, Mikolajczyk R, et al. *Social contacts and mixing patterns relevant to the spread of infectious diseases.* PLoS Med, 2008. **5**(3): p. e74.

3. Szusz EK, Garrison LP, Bauch CT. *A review of data needed to parameterize a dynamic model of measles in developing countries.* BMC Res Notes, 2010. **3**: p. 75.

4. Wallinga J, Levy-Bruhl D, Gay NJ, Wachmann CH. *Estimation of measles reproduction ratios and prospects for elimination of measles by vaccination in some Western European countries.* Epidemiol Infect, 2001. **127**(2): p. 281-95.

5. Miller MA. *Introducing a novel model to estimate national and global measles disease burden.* Int J Infect Dis, 2000. **4**(1): p. 14-20.

6. Organization WH, *Measles Strategic Planning Tool. Version 1.2.* . 2008: Geneva, New York.

7. Heffernan JM, Keeling MJ. *Implications of vaccination and waning immunity.* 2009, Proceedings of the Royal Society of London B: Biological Sciences.

8. Division UNP. *World Population Prospects: The 2006 Revision*. 2007, United Nations: New York.

9. Wolfson LJ, Gasse F, Lee-Martin SP, Lydon P, Magan A, Tibouti A, et al. *Estimating the costs of achieving the WHO-UNICEF Global Immunization Vision and Strategy, 2006-2015.* Bull World Health Organ, 2008. **86**(1): p. 27-39.

10. Beutels P, Gay NJ. *Economic evaluation of options for measles vaccination strategy in a hypothetical Western European country.* Epidemiol Infect, 2003. **130**(2): p. 273-83.

11. Levin A, England S, Jorissen J, Garshong B, Teprey J. *Case study on the costs and financing of immunization services in Ghana*, in *Partners in Health Reform*. 2001, Abt Associates: Bethesda, MD.

12. Walker DG, Hutubessy R, Beutels P. *WHO Guide for standardisation of economic evaluations of immunization programmes.* Vaccine, 2010. **28**(11): p. 2356-9.

13. Edejer, TTT. *Making choices in health: WHO guide to cost-effectiveness analysis*. 2003, Geneva: World Health Organization. 329.

14. Orenstein WA, Markowitz L, Preblud SR, Hinman AR, Tomasi A, Bart KJ *Appropriate age for measles vaccination in the United States.* Dev Biol Stand, 1986. **65**: p. 13-21.

15. Alonso WJ, Rabaa MA, Giglio R, Balinska M, Miller, MA. *Decision Analytical Support for Strategies for Multiple Doses of Measles Vaccine.* In: 2010 International Conference on Emerging Infectious Diseases, 2010, Atlanta. International Conference on Emerging Infectious Diseases - Program and Abstracts Book, 2010. p. 219-219.

### Table A. General model assumptions, epidemiological and cost parameters.

| Parameter | Description | Source |
| --- | --- | --- |
| Age cohorts | 5 sub-year groups (<3; 3; 4-11; 12-14; 15-23 months); 98 year groups (from 3-99 years); 1 group of 100+ years |  |
| Vaccination coverage | Country-specific data.  Based on single age-groups (MCV1 and MCV2)  or range of age-groups (SIAs) | Dabbagh, A., Strebel, P., Gacic –Dobo, M. *pers. communication* (2008) |
| Vaccine efficacy | 0% for vaccination for 0 to <3 months  65% for vaccination for 3 months  85% for vaccination for 4 to <12 months  95% for vaccination for 12+ months | [15] |
| Maternal antibody immunity | 100% for 0 to <3 months  30% for 3 months  10% for 4 to <12 months  0% for 12+ months | [16] |
| Force of infection in static and semi-dynamic models | \| Age (years) \| Low income \| High income \| \| --- \| --- \| --- \| \| <1 \| 0.121 \| 0.121 \| \| 1 to <5 \| 0.333 \| 0.121 \| \| 5 to <10 \| 0.345 \| 0.345 \| \| 10 to <15 \| 0.201 \| 0.201 \| \| 15 to <20 \| 0.144 \| 0.144 \| \| 20+ \| 0.068 \| 0.068 \| | [5, 17] |
| Stochastic immigration factor* | Remote countries† = 7.37 x 10-7  Non-remote countries† = 2.55 x 10-5 | [5, 18] |
| Case fatality rates | Country-specific (range: 0.05% to 6%) × age-specific multipliers:  2 for <1 year olds  1 for 1 to <4 years  0.5 for 5 to <10 years  0 for +10 years | [8] |

^*^Applied to those countries that effectively reduced population susceptibility to <6.7%.

^†^ Countries: Argentina, Belize, Bolivia, Canada, Chile, Costa Rica, Ecuador, El Salvador, Guatemala, Honduras, Mexico, Nicaragua, Panama, Peru, Uruguay, and the United States, Venezuela; island countries include the Bahamas, Barbados, Cuba, Grenada, Guyana, Haiti, Jamaica, and Trinidad & Tobago.

## Table B. Measles morbidity and mortality in India under each immunization scenario (S1-S4; see Fig.2 in the main manuscript) as estimated with the dynamic transmission model. Values represent the number of cases and deaths from 2008 to 2050. ICER: incremental cost-effectiveness ratio (US$/DALY averted) using S1 as baseline. Per capita GDP in India (UN, 2010): US$ 1,061. MCV1 coverage in 2006 is indicated below the name of the Indian state.

| **Indian State** | **Estimate** | **S1** | **S2** | **S3** | **S4** |
| --- | --- | --- | --- | --- | --- |
| Bihar | Cases | 68,776,673 | 36,180,148 | 44,906,078 | 3,542,853 |
| (MCV1: 46%) | Deaths | 518,232 | 206,527 | 281,850 | 19,266 |
|  | DALY | 3,271,157 | 1,701,583 | 2,054,760 | 222,164 |
|  | Costs | $7,020,285 | $13,448,784 | $14,374,235 | $64,668,175 |
|  | ICER |  | $4.10** | $6.05** | $18.91** |
| Karnataka | Cases | 12,444,501 | 12,444,501 | 5,541,908 | 2,012,247 |
| (MCV1: 90%) | Deaths | 39,920 | 39,920 | 3,021 | 2,870 |
|  | DALY | 399,712 | 399,712 | 127,330 | 59,466 |
|  | Costs | $5,961,300 | $5,961,300 | $12,221,211 | $32,362,056 |
|  | ICER |  | * | $22.98** | $77.59** |
| Maharashtra | Cases | 34,598,948 | 24,493,778 | 14,956,073 | 3,678,120 |
| (MCV1: 74%) | Deaths | 168,781 | 93,263 | 12,677 | 6,522 |
|  | DALY | 1,314,426 | 856,622 | 314,667 | 109,989 |
|  | Costs | $12,495,391 | $15,133,448 | $25,692,433 | $55,712,049 |
|  | ICER |  | $5.76** | $13.20** | $35.88** |
| Orissa | Cases | 10,093,134 | 9,181,174 | 4,464,550 | 1,414,647 |
| (MCV1: 86%) | Deaths | 41,240 | 35,031 | 7,146 | 3,387 |
|  | DALY | 373,696 | 335,489 | 137,305 | 52,038 |
|  | Costs | $4,969,791 | $5,201,439 | $10,141,937 | $25,980,251 |
|  | ICER |  | $6.06** | $21.88** | $65.32** |
| Tamil Nadu | Cases | 9,418,944 | 9,418,944 | 4,305,989 | 2,012,509 |
| (MCV1: 95%) | Deaths | 23,018 | 23,018 | 94 | 1,602 |
|  | DALY | 274,519 | 274,519 | 80,866 | 50,146 |
|  | Costs | $6,253,172 | $6,253,172 | $12,825,641 | $31,868,963 |
|  | ICER |  | * | $33.94** | $114.17** |

## *Cost of S1 and S2 is the same: differences in cost-effectiveness represented by DALY differences

** Cost-effective strategy (ICER lower than per capita GDP of the country)

## Table C. Measles morbidity and mortality in India under each immunization scenario (S1-S4) as estimated with the static transmission model. Values represent the total number of cases and deaths from 2008 to 2050. ICER: incremental cost-effectiveness ratio, in US$/DALY averted, using S1 as baseline. Per capita GDP in India (UN, 2010): US$ 1,061. MCV1 coverage in 2006 is indicated below the name of the Indian state.

| **Indian State** | **Estimate** | **S1** | **S2** | **S3** | **S4** |
| --- | --- | --- | --- | --- | --- |
| Bihar | Cases | 21,176,311 | 10,432,582 | 13,713,030 | 1,735,933 |
| (MCV1: 46%) | Deaths | 319,434 | 148,332 | 208,483 | 35,626 |
|  | DALY | 1,838,886 | 953,823 | 1,251,719 | 248,618 |
|  | Costs | $7,020,285 | $13,448,784 | $1,4374,235 | $64,668,175 |
|  | ICER |  | $7.26** | $12.52** | $36.25** |
| Karnataka | Cases | 3,434,177 | 3,434,177 | 1,193,473 | 923,234 |
| (MCV1: 90%) | Deaths | 51,493 | 51,493 | 5,399 | 17,137 |
|  | DALY | 347,538 | 347,538 | 57,742 | 122,575 |
|  | Costs | $5,961,300 | $5,961,300 | $12,221,211 | $32,362,056 |
|  | ICER |  | * | $21.60** | $117.35** |
| Maharashtra | Cases | 1,1621,792 | 7,929,676 | 4,859,358 | 2,186,108 |
| (MCV1: 74%) | Deaths | 188,965 | 126,053 | 25,610 | 45,215 |
|  | DALY | 1,228,111 | 852,919 | 230,666 | 312,740 |
|  | Costs | $12,495,391 | $15,133,448 | $25,692,433 | $55,712,049 |
|  | ICER |  | $7.03** | $13.23** | $47.21** |
| Orissa | Cases | 3,106,973 | 2,783,888 | 1,169,040 | 829,819 |
| (MCV1: 86%) | Deaths | 48,391 | 42,921 | 11,214 | 16,239 |
|  | DALY | 322,981 | 290,557 | 93,835 | 114,200 |
|  | Costs | $4,969,791 | $5,201,439 | $10,141,937 | $25,980,251 |
|  | ICER |  | $7.14** | $22.57** | $100.63** |
| Tamil Nadu | Cases | 2,669,410 | 2,669,410 | 847,091 | 803,456 |
| (MCV1: 95%) | Deaths | 40,239 | 40,239 | 248 | 14,382 |
|  | DALY | 296,346 | 296,346 | 17,665 | 112,090 |
|  | Costs | $6,253,172 | $6,253,172 | $12,825,641 | $31,868,963 |
|  | ICER |  | * | $23.58** | $139.02** |

## *Cost of S1 and S2 is the same: differences in cost-effectiveness represented by DALY differences

** Cost-effective strategy (ICER lower than per capita GDP of the country)

## Table D. Measles morbidity and mortality in India under each immunization scenario (S1-S4) as estimated with the semi-dynamic transmission model. Values represent the number of cases and deaths from 2008 to 2050. ICER: incremental cost-effectiveness ratio, in US$/DALY averted, using S1 as baseline. Per capita GDP in India (UN, 2010): US$ 1,061. MCV1 coverage in 2006 is indicated below the name of the Indian state.

| **Indian State** | **Estimate** | **S1** | **S2** | **S3** | **S4** |
| --- | --- | --- | --- | --- | --- |
| Bihar | Cases | 30,051,075 | 16,679,794 | 21,289,183 | 799,450 |
| (MCV1: 46%) | Deaths | 358,856 | 164,198 | 227,849 | 8,798 |
|  | DALY | 2,100,791 | 1,095,670 | 1,407,320 | 93,589 |
|  | Costs | $7,020,285 | $13,448,784 | $14,374,235 | $64,668,175 |
|  | ICER |  | $6.40** | $10.60** | $28.72** |
| Karnataka | Cases | 6,301,616 | 6,301,616 | 1,829,249 | 528,197 |
| (MCV1: 90%) | Deaths | 56,861 | 56,861 | 3,732 | 3,719 |
|  | DALY | 405,069 | 405,069 | 61,947 | 34,706 |
|  | Costs | $5,961,300 | $5,961,300 | $12,221,211 | $32,362,056 |
|  |  |  | * | $18** | $71** |
| Maharashtra | Cases | 21,645,136 | 15,328,207 | 8,424,377 | 1,453,497 |
| (MCV1: 74%) | Deaths | 61,469 | 38,203 | 22,044 | 2,788 |
|  | DALY | 631,326 | 436,808 | 258,037 | 45,933 |
|  | Costs | $12,495,391 | $15,133,448 | $25,692,433 | $55,712,049 |
|  |  |  | $14** | $35** | $74** |
| Orissa | Cases | 5,834,524 | 5,281,872 | 1,742,873 | 497,563 |
| (MCV1: 86%) | Deaths | 14,919 | 12,972 | 8,084 | 1,035 |
|  | DALY | 166,057 | 149,152 | 86,581 | 16,271 |
|  | Costs | $4,969,791 | $5,201,439 | $10,141,937 | $25,980,251 |
|  |  |  | $14** | $65** | $140** |
| Tamil Nadu | Cases | 5,686,000 | 5,686,000 | 1,089,214 | 597,650 |
| (MCV1: 95%) | Deaths | 11,688 | 11,688 | 134 | 885 |
|  | DALY | 153,638 | 153,638 | 23,228 | 19,369 |
|  | Costs | $6,253,172 | $6,253,172 | $12,825,641 | $31,868,963 |
|  |  |  | * | $50.39** | $190.77** |

## *Cost of S1 and S2 is the same: differences in cost-effectiveness represented by DALY differences

** Cost-effective strategy (ICER lower than per capita GDP of the country)

## Table E. Measles morbidity and mortality in Africa and Cambodia under each immunization scenario (S1-S3; see Fig.2 in the main manuscript) as estimated with the dynamic model. Values represent the total number of cases and deaths from 2008 to 2050. ICER: incremental cost-effectiveness ratio (US$/DALY averted) using S1 as baseline. Per capita GDP in Cameroon, Democratic Republic of Congo, Equatorial Guinea, Ghana, Rwanda and Cambodia, respectively (UN, 2010): US$ 1,217; US$180; US$27,130; US$709; US$458 and US$768. MCV1 coverage in 2006 is indicated below the name of the country.

| **Country** | **Estimate** | **S1** | **S2** | **S3** |
| --- | --- | --- | --- | --- |
| Cameroon | Cases | 1,234,404 | 792,140 | 1,137,300 |
| (MCV1: 73%) | Deaths | 49,181 | 36,583 | 48,682 |
|  | DALY | 228,373 | 164,127 | 225,099 |
|  | Costs | $45,686,073 | $65,025,218 | $69,958,590 |
|  | ICER |  | $301** | $7,414 |
| Democratic Rep. | Cases | 8,780,860 | 5,575,937 | 8,157,168 |
| Congo | Deaths | 447,569 | 331,536 | 443,256 |
| (MCV1: 73%) | DALY | 1,498,929 | 1,052,293 | 1,475,734 |
|  | Costs | $167,914,509 | $236,934,935 | $250,278,457 |
|  | ICER |  | $155** | $3,551 |
| Equatorial Guinea | Cases | 46,376 | 40,741 | 40,857 |
| (MCV1: 51%) | Deaths | 2,151 | 1,976 | 2,117 |
|  | DALY | 9,289 | 8,473 | 9,018 |
|  | Costs | $2,385,641 | $3,143,839 | $3,286,008 |
|  | ICER |  | $929** | $3,322** |
| Ghana | Cases | 1,497,263 | 804,144 | 1,356,877 |
| (MCV1: 85%) | Deaths | 73,601 | 44,904 | 72,753 |
|  | DALY | 380,773 | 224,715 | 374,926 |
|  | Costs | 53,563,457 | $75,653,817 | $81,832,606 |
|  | ICER |  | $142** | $4,835 |
| Rwanda | Cases | 740,127 | 364,351 | 686,159 |
| (MCV1: 95%) | Deaths | 32,806 | 19,707 | 32,507 |
|  | DALY | 29,575,426 | 42,759,204 | 45,229,059 |
|  | Costs | $132,850 | $74,644 | $131,005 |
|  | ICER |  | $227** | $8,484 |
| Cambodia | Cases | 847,520 | 491,955 | 789,667 |
| (MCV1: 78%) | Deaths | 19,056 | 12,111 | 18,914 |
|  | DALY | 109,649 | 67,836 | 108,245 |
|  | Costs | $30,018,029 | $41,278,994 | $44,537,377 |
|  | ICER |  | $269** | $10,341 |

** Cost-effective strategy (ICER lower than per capita GDP of the country)

Table F. Measles morbidity and mortality in Africa and Cambodia under each immunization scenario (S1-S3) as estimated with the static model. Values represent the total number of cases and deaths from 2008 to 2050. ICER: incremental cost-effectiveness ratio, in US$/DALY averted, using S1 as baseline. Per capita GDP in Cameroon, Democratic Rep. of Congo, Equatorial Guinea, Ghana, Rwanda and Cambodia, respectively (UN, 2010): US$ 1,217; US$180; US$27,130; US$709; US$458 and US$768. MCV1 coverage in 2006 is indicated below the name of the country.

| **Country** | **Estimate** | **S1** | **S2** | **S3** |
| --- | --- | --- | --- | --- |
| Cameroon | Cases | 1,896,743 | 1,013,036 | 1,859,822 |
| (MCV1: 73%) | Deaths | 77,168 | 47,687 | 76,673 |
|  | DALY | 368,186 | 219,479 | 365,571 |
|  | Costs | $45,686,073 | $65,025,218 | $69,958,590 |
|  | ICER |  | $130** | $9,282 |
| Democratic Rep. | Cases | 12,468,837 | 6,549,210 | 12,207,042 |
| Congo | Deaths | 607,781 | 370,416 | 603,540 |
| (MCV1: 73%) | DALY | 2,139,471 | 1,239,524 | 2,120,532 |
|  | Costs | $167,914,509 | $236,934,935 | $250,278,457 |
|  | ICER |  | $77** | $4,349 |
| Equatorial | Cases | 56,951 | 45,569 | 55,291 |
| Guinea | Deaths | 2,850 | 2,423 | 2,825 |
| (MCV1: 51%) | DALY | 12,605 | 10,681 | 12,442 |
|  | Costs | $2,385,641 | $3,143,839 | $3,286,008 |
|  | ICER |  | $394** | $5,524** |
| Ghana | Cases | 2,495,281 | 1,026,604 | 2,448,822 |
| (MCV1: 85%) | Deaths | 137,315 | 66,484 | 136,433 |
|  | DALY | 724,645 | 341,982 | 719,670 |
|  | Costs | $53,563,457 | $75,653,817 | $81,832,606 |
|  | ICER |  | $58** | $5,682 |
| Rwanda | Cases | 1,220,948 | 402,815 | 1,198,637 |
| (MCV1: 95%) | Deaths | 54,184 | 22,527 | 53,841 |
|  | DALY | 229,231 | 89,588 | 227,573 |
|  | Costs | $29,575,426 | $42,759,204 | $45,229,059 |
|  | ICER |  | $94** | $9,441 |
| Cambodia | Cases | 1,519,800 | 708,886 | 1,501,221 |
| (MCV1: 78%) | Deaths | 39,114 | 20,948 | 3,8952 |
|  | DALY | 225,943 | 117,966 | 224,880 |
|  | Costs | $30,018,029 | $41,278,994 | $44,537,377 |
|  | ICER |  | $104** | $13,659 |

** Cost-effective strategy (ICER lower than per capita GDP of the country)

Table G. Measles morbidity and mortality in Africa and Cambodia under each immunization scenario (S1-S3) as estimated with the semi-dynamic model. Values represent the total number of cases and deaths from 2008 to 2050. ICER: incremental cost-effectiveness ratio, in US$/DALY averted, using S1 as baseline. Per capita GDP in Cameroon, Democratic Rep. Congo, Equatorial Guinea, Ghana, Rwanda and Cambodia (UN, 2010): US$ 1,217; US$180; US$27,130; US$709; US$458 and US$768, respectively. MCV1 coverage in 2006 is given below the name of the country.

| **Country** | **Estimate** | **S1** | **S2** | **S3** |
| --- | --- | --- | --- | --- |
| Cameroon | Cases | 561,441 | 46,146 | 212,137 |
| (MCV1: 73%) | Deaths | 14,319 | 1,231 | 6,258 |
|  | DALY | 69,977 | 6,238 | 31,337 |
|  | Costs | $45,686,073 | $65,025,218 | $69,958,590 |
|  | ICER |  | $303** | $628** |
| Democratic Rep. | Cases | 4,292,048 | 541,818 | 2,036,828 |
| Congo | Deaths | 140,054 | 17,784 | 77,221 |
| (MCV1: 73%) | DALY | 549,275 | 76,200 | 331,382 |
|  | Costs | $167,914,509 | $236,934,935 | $250,278,457 |
|  | ICER |  | $146** | $378 |
| Equatorial Guinea | Cases | 20,886 | 14,501 | 13,692 |
| (MCV1: 51%) | Deaths | 683 | 509 | 517 |
|  | DALY | 4,526 | 3,657 | 3,688 |
|  | Costs | $2,385,641 | $3,143,839 | $3,286,008 |
|  | ICER |  | $872** | $1,074** |
| Ghana | Cases | 1,022,306 | 65,442 | 519,065 |
| (MCV1: 85%) | Deaths | 40,650 | 2,403 | 23,452 |
|  | DALY | 222,261 | 16459 | 137,013 |
|  | Costs | $53,563,457 | $75,653,817 | $81,832,606 |
|  | ICER |  | $107** | $332** |
| Rwanda | Cases | 332,027 | 12,644 | 190,440 |
| (MCV1: 95%) | Deaths | 10,554 | 435 | 7,296 |
|  | DALY | 48,250 | 2,759 | 33,062 |
|  | Costs | $29,575,426 | $42,759,204 | $45,229,059 |
|  | ICER |  | $290** | $1,031 |
| Cambodia | Cases | 518,588 | 36,792 | 349,529 |
| (MCV1: 78%) | Deaths | 9,963 | 664 | 7,457 |
|  | DALY | 60,624 | 4,924 | 47,282 |
|  | Costs | $30,018,029 | $41,278,994 | $44,537,377 |
|  | ICER |  | $202** | $1,088 |

** Cost-effective strategy (ICER lower than per capita GDP of the country)

## Table H. Measles morbidity and mortality in Latin American under each immunization scenario (S1-S4; Fig.2 in the manuscript) as estimated with the dynamic transmission model. Values represent the total number of cases and deaths from 2008-2050. ICER: incremental cost-effectiveness ratio, in US$/DALY averted, using S1 as baseline. Here, ICERs must be interpreted as the cost saved per DALY averted (higher ICERs are better). Per capita GDP in Costa Rica, El Salvador, Paraguay and Mexico, respectively (UN, 2010): US$ 6,599; US$ 3,605; US$ 2,581 and US$ 9,964.

| **Country** | **Estimate** | **S1** | **S2** | **S3** | **S4** |
| --- | --- | --- | --- | --- | --- |
| Costa Rica | Cases | 209,631 | 206,915 | 402,210 | 387,959 |
| (MCV1: 89%) | Deaths | 123 | 123 | 216 | 215 |
|  | DALY | 4,117 | 4,067 | 7,509 | 7,317 |
|  | Costs | $14,958,756 | $14,958,756 | $9,381,646 | $9,381,646 |
|  | ICER |  | * | $1644.20 | $1742.85 |
| El Salvador | Cases | 277,705 | 277,662 | 463,734 | 462,480 |
| (MCV1: 98%) | Deaths | 210 | 210 | 311 | 310 |
|  | DALY | 5,440 | 5,439 | 9,010 | 8,988 |
|  | Costs | $25,294,879 | $25,294,879 | $12,653,182 | $12,653,182 |
|  | ICER |  | * | $3,541 | $3,563 |
| Paraguay | Cases | 352,166 | 344,764 | 716,604 | 612,346 |
| (MCV1: 88%) | Deaths | 254 | 253 | 426 | 409 |
|  | DALY | 6,854 | 6,711 | 12,859 | 11,368 |
|  | Costs | $16,420,915 | $16,420,915 | $7,536,687 | $7,536,687 |
|  | ICER |  | * | $1,479** | $1,968 |
| Mexico | Cases | 2,791,174 | 2,780,767 | 5,297,368 | 4,998,231 |
| (MCV1: 96%) | Deaths | 2,345 | 2,344 | 3,663 | 3,638 |
|  | DALY | 59,224 | 59,057 | 103,190 | 99,367 |
|  | Costs | $262,625,378 | $262,625,378 | $123,061,389 | $123,061,389 |
|  | ICER |  | * | $3,174 | $3,476 |

## *Cost of S1 and S2 is the same (no cost to target unvaccinated individuals): differences in cost-effectiveness represented by DALY differences

## Table I. Measles morbidity and mortality in Latin American under each immunization scenario (S1-S4) as estimated with the static transmission model. Values represent the total number of cases and deaths from 2008 to 2050. ICER: incremental cost-effectiveness ratio, in US$/DALY averted, using S1 as baseline. Here, ICERs must be interpreted as the cost saved per DALY averted (higher ICERs are better). Per capita GDP in Costa Rica, El Salvador, Paraguay and Mexico, respectively (UN, 2010): US$ 6,599; US$ 3,605; US$ 2,581 and US$ 9,964.

| **Country** | **Estimate** | **S1** | **S2** | **S3** | **S4** |
| --- | --- | --- | --- | --- | --- |
| Costa Rica | Cases | 310,949 | 310,247 | 604,386 | 600,427 |
| (MCV1: 89%) | Deaths | 211 | 211 | 359 | 358 |
|  | DALY | 6,241 | 6,228 | 11,503 | 11,430 |
|  | Costs | $14,958,756 | $14,958,756 | $9,381,646 | $9,381,646 |
|  | ICER |  | * | $1,060 | $1,075 |
| El Salvador | Cases | 333,598 | 333,545 | 538,926 | 537,759 |
| (MCV1: 98%) | Deaths | 280 | 280 | 410 | 409 |
|  | DALY | 6,560 | 6,552 | 10,447 | 10,431 |
|  | Costs | $25,294,879 | $25,294,879 | $12,653,182 | $12,653,182 |
|  | ICER |  | * | $3,252 | $3,266 |
| Paraguay | Cases | 471,527 | 466,246 | 961,434 | 887,185 |
| (MCV1: 88%) | Deaths | 349 | 347 | 603 | 579 |
|  | DALY | 9,263 | 9,134 | 17,715 | 16,506 |
|  | Costs | $16,420,915 | $16,420,915 | $7,536,687 | $7,536,687 |
|  | ICER |  | * | $1,051 | $1,227 |
| Mexico | Cases | 4,588,266 | 4,582,737 | 8,451,180 | 8,330,987 |
| (MCV1: 96%) | Deaths | 3,653 | 3,652 | 5,739 | 5,713 |
|  | DALY | 96,898 | 96,794 | 167,520 | 165,679 |
|  | Costs | $262,625,378 | $262,625,378 | $123,061,389 | $123,061,389 |
|  | ICER |  | * | $1,976 | $2,029 |

## *Cost of S1 and S2 is the same (no cost to target unvaccinated individuals): differences in cost-effectiveness represented by DALY differences

## Table J. Measles morbidity and mortality for each immunization scenario (S1-S4) in Latin American as estimated with the semi-dynamic transmission model. Values represent the total number of cases and deaths from 2008 to 2050. ICER: incremental cost-effectiveness ratio, in US$/DALY averted, using S1 as baseline. Here, ICERs must be interpreted as the cost saved per DALY averted (higher ICERs are better). Per capita GDP in Costa Rica, El Salvador, Paraguay and Mexico, respectively (UN, 2010): US$ 6,599; US$ 3,605; US$ 2,581 and US$ 9,964.

| **Country** | **Estimate** | **S1** | **S2** | **S3** | **S4** |
| --- | --- | --- | --- | --- | --- |
| Costa Rica | Cases | 78,338 | 72,820 | 425,251 | 394,977 |
| (MCV1: 89%) | Deaths | 34 | 32 | 202 | 191 |
|  | DALY | 1,716 | 1,610 | 7,879 | 7,360 |
|  | Costs | $14,958,756 | $14,958,756 | $9,381,646 | $9,381,646 |
|  | ICER |  | * | $905 | $988 |
| El Salvador | Cases | 152 | 152 | 26,009 | 25,700 |
| (MCV1: 98%) | Deaths | 0 | 0 | 13 | 13 |
|  | DALY | 1 | 1 | 439 | 439 |
|  | Costs | $25,294,879 | $25,294,879 | $12,653,182 | $12,653,182 |
|  | ICER |  | * | $28,862 | $28,862 |
| Paraguay | Cases | 57,709 | 44,122 | 590,180 | 377,673 |
| (MCV1: 88%) | Deaths | 26 | 21 | 264 | 189 |
|  | DALY | 1,418 | 1,131 | 10,149 | 6,780 |
|  | Costs | $16,420,915 | $16,420,915 | $7,536,687 | $7,536,687 |
|  | ICER |  | * | $1,018 | $1,657 |
| Mexico | Cases | 28,632 | 27,698 | 3,515,889 | 2,231,841 |
| (MCV1: 96%) | Deaths | 12 | 12 | 1,524 | 1,011 |
|  | DALY | 640 | 616 | 57,956 | 37,630 |
|  | Costs | $262,625,378 | $262,625,378 | $123,061,389 | $123,061,389 |
|  | ICER |  | * | $2,435 | $3,773 |

## *Cost of S1 and S2 is the same (no cost to target unvaccinated individuals): differences in cost-effectiveness represented by DALY differences

Table K. Results of the sensitivity analyses: estimated morbidity and mortality from measles in Equatorial Guinea, Tamil Nadu and Costa Rica from 2008 to 2050 under extreme values of key epidemiological and cost parameters (lower and upper bound: best and worst case scenarios, respectively). For Costa Rica, ICERs should be interpreted as the cost saved per DALY averted**.**

| Country |  | Scenario | Cases | Deaths | DALY | Costs | ICER* |
| --- | --- | --- | --- | --- | --- | --- | --- |
| Equatorial | Lower Bound | S1 | 29,858 | 690 | 696 | $1,184,509 |  |
| Guinea |  | S2 | 25,447 | 650 | 652 | $1,570,528 | $8,773 |
|  |  | S3 | 27,346 | 677 | 661 | $1,626,397 | $12,625 |
|  | Upper Bound | S1 | 74,538 | 4,898 | 85,125 | $3,553,528 |  |
|  |  | S2 | 63,447 | 4,405 | 75,100 | $4,669,868 | $111 |
|  |  | S3 | 68,127 | 4,833 | 83,449 | $4,879,192 | $790 |
| Tamil Nadu | Lower Bound | S1 | 5,317,273 | 76 | 17,470 | $3,132,270 |  |
|  |  | S2 | 5,317,273 | 76 | 17,470 | $3,132,270 | ** |
|  |  | S3 | 2,965,566 | 16 | 13,701 | $6,424,479 | $873 |
|  |  | S4 | 1,544,456 | 5 | 7,741 | $15,917,545 | $1,314 |
|  | Upper Bound | S1 | 6,945,496 | 2,037 | 474,046 | $9,396,811 |  |
|  |  | S2 | 6,945,496 | 2,037 | 474,046 | $9,396,811 | ** |
|  |  | S3 | 2,492,637 | 537 | 165,095 | $19,273,438 | $32 |
|  |  | S4 | 1,419,876 | 325 | 94,780 | $47,752,635 | $101 |
| Costa Rica | Lower Bound | S1 | 170,994 | 52 | 633 | $7,463,377 |  |
|  |  | S2 | 168,187 | 52 | 627 | $7,463,377 | ** |
|  |  | S3 | 325,324 | 92 | 1,072 | $4,682,892 | $6,334 |
|  |  | S4 | 311,356 | 92 | 1,098 | $4,682,892 | $5,980 |
|  | Upper Bound | S1 | 174,181 | 118 | 13,618 | $22,463,683 |  |
|  |  | S2 | 172,591 | 118 | 13,507 | $22,463,683 | ** |
|  |  | S3 | 688,410 | 493 | 55,875 | $14,122,230 | $197 |
|  |  | S4 | 674,186 | 485 | 54,705 | $14,122,230 | $202 |

*ICER: incremental cost-effectiveness ratio, in US$/DALY averted, using S1 as baseline.

## **The cost of S1 and S2 is the same (differences in cost-effectiveness are represented by DALY differences)
